# Cardiac magnetic resonance imaging using an open 1.0T MR platform: a comparative study with 1.5T

**DOI:** 10.1186/1532-429X-15-S1-E41

**Published:** 2013-01-30

**Authors:** Ortrud Kosiek, Katharina A Strach, Bernhard Schnackenburg, Alexander Schmeisser, Jan Smid, Friederike Walz, Jens Ricke, Frank Fischbach

**Affiliations:** 1 Department of Radiology and Nuclear Medicine, University Magdeburg, Magdeburg, Germany; 2 Department of Cardiology, University Magdeburg, Magdeburg, Germany; 3 Clinical Science, Philips Healthcare, Hamburg, Germany

## Background

Cardiac MRI (cMRI) has evolved as the non-invasive reference standard for accurate and highly reproducible determination of cardiac function and myocardial viability. Thus, cMRI is widespread and increasingly utilized in the clinical practice. The introduction of open high-field (HFO) MR scanners offers advantages not only for image-guided interventions, examinations of patients with claustrophobia or children, but also for monitoring of critically ill patients.

Therefore, the aim of our study was to evaluate unenhanced cMR imaging sequences such as cine balanced-steady-state-free-precession (B-SSFP), T1-weighted (w) turbo spin echo (TSE), T1-w Black-Blood (BB) turbo field echo (TFE), and T2-w BB TSE sequences acquired at an open 1.0T MR scanner with regard to 1) image quality and 2) amount of non-diagnostic (ND) myocardial segments (S) compared to a standard cylindrical 1.5T MR system.

**Figure 1:**
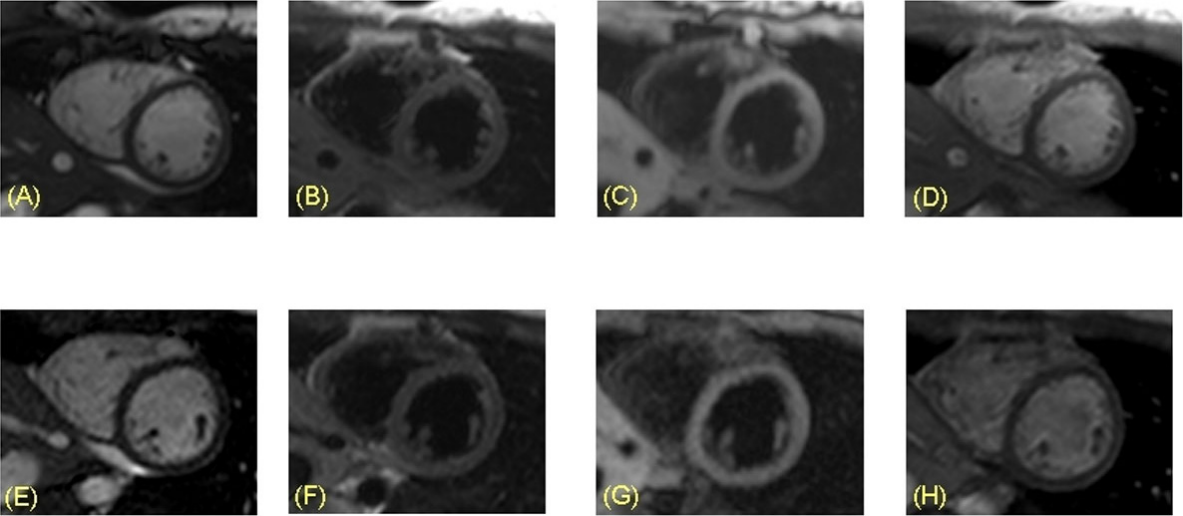
B-SSFP (A+E), T2 BB TSE (B+F), T1 BB TFE (C+G) and T1 TSE (D+H) sequences acquired both with a standard cylindrical 1.5T MR scanner (A-D) and an open 1.0T MR system (E-H) in the same healthy volunteer. Image quality was rated 4 (excellent) for both field strengths.

## Methods

15 volunteers (9 female, 6 male; 36+/- 17 years) underwent unenhanced cMRI both at an open 1.0T MR scanner (Panorama HFO 1.0T, Philips Healthcare, Best, Netherlands) and a cylindrical 1.5T MR system (Intera 1.5T, Philips Healthcare, Best, Netherlands). Cine B-SSFP, T1 TSE, T1 TFE and T2 TSE sequences in three [apical (a), mid-ventricular (m), basal (b)] short axis slices (SA) were acquired at both field strengths. Overall image quality using a 4-point grading scale (4: excellent; 1: non-diagnostic) was assessed. For semiquantitative assessment of artifacts 16 myocardial S were evaluated for each of the four different sequence types. The location and total number of ND segments was determined.

## Results

Image quality did not show a statistically significant difference for all four sequence types both at 1.0T (B-SSFP 3.87±0.35; T1 TSE 3.13±0.74; T1 BB TFE 3.6±0.63; T2 BB TSE 3.6±0.63) and 1.5T (B-SSFP 3.67±0.49; T1 TSE 3.73±0.46; T1 BB TFE 3.33±0.49; T2 BB TSE 3.47±0.52; p > 0.05). ND myocardial S occurred only in the T2 TSE sequence (6/240 Segments, 2.5%) using the HFO platform. At the cylindrical 1.5T scanner 8/240 (3.3%) segments were non-diagnostic for the T2 TSE and 9/240 (3.8%) segments for the T1 TFE. The majority of ND S at both field strengths was detected in the b SA slices [1.0T: T2 TSE 6 segments (b 4/6 segments: 2/4 inferolateral, 1/4 anterolateral, 1/4 inferoseptal; m 2/6 segments: 2/2 inferolateral); 1.5T: T2 TSE 8 segments (b 6/8 segments - 2/6 inferolateral, 3/6 anterolateral, 1/6 inferior; m 2/8 segments - 1/2 inferolateral, 1/2 inferior), T1 TFE 9 segments (b 7/9 segments - 4/7 inferolateral, 1/7 anterolateral, 2/7 inferior; m 2/9 segments: 2/2 anterolateral)].

## Conclusions

Standard cardiac MRI sequences at an open 1.0T MR platform offer a high image quality, which is comparable to sequences acquired at a cylindrical 1.5T MR scanner. The number of non-diagnostic myocardial segments are reduced at an open 1.0T MR system when compared to the standard approach at 1.5T.

## Funding

None.

